# Forecasting Energy Demand in Quicklime Manufacturing: A Data-Driven Approach

**DOI:** 10.3390/s25247632

**Published:** 2025-12-16

**Authors:** Jersson X. Leon-Medina, John Erick Fonseca Gonzalez, Nataly Yohana Callejas Rodriguez, Mario Eduardo González Niño, Saúl Andrés Hernández Moreno, Wilman Alonso Pineda-Munoz, Claudia Patricia Siachoque Celys, Bernardo Umbarila Suarez, Francesc Pozo

**Affiliations:** 1 Grupo de Investigación en Biochar, Suelo y Cambio Climático (Pyrosfera), Suministros Mineros e Industriales de Colombia LTDA-SUMININCO LTDA, Km1 vía Nobsa-Duitama Vereda Guaquida, Nobsa 152280, Colombia; cpsiachoque@gmail.com (C.P.S.C.); bernardoumbarila@gmail.com (B.U.S.); 2 Grupo de Investigación en Energía y Nuevas Tecnologías—GENTE, Escuela de Ingeniería Electromecánica, Facultad Seccional Duitama, Universidad Pedagógica y Tecnológica de Colombia, Carrera 18 con Calle 22, Duitama 150461, Colombia; johnfonsecagonzalez@gmail.com (J.E.F.G.); marioeduardo.gonzalez@uptc.edu.co (M.E.G.N.); saul.hernandez@uptc.edu.co (S.A.H.M.); wilman.pineda@uptc.edu.co (W.A.P.-M.); 3 Especialización de Sistemas de Información Geográfica, Fundación Universitaria Juan de Castellanos, Tunja 150003, Colombia; natycallejas05@gmail.com; 4 Control, Data, and Artificial Intelligence (CoDAlab), Department of Mathematics, Escola d’Enginyeria de Barcelona Est (EEBE), Campus Diagonal-Besòs (CDB), Universitat Politècnica de Catalunya (UPC), Eduard Maristany 16, 08019 Barcelona, Spain; francesc.pozo@upc.edu; 5 Institute of Mathematics (IMTech), Universitat Politècnica de Catalunya (UPC), Pau Gargallo 14, 08028 Barcelona, Spain

**Keywords:** energy consumption prediction, recurrent neural network, deep learning, Long Short-Term Memory (LSTM), Gated Recurrent Unit (GRU), time series forecasting

## Abstract

This study presents a deep learning-based framework for forecasting energy demand in a quicklime production company, aiming to enhance operational efficiency and enable data-driven decision-making for industrial scalability. Using one year of real electricity consumption data, the methodology integrates temporal and operational variables—such as load profile, active power, shift indicators, and production-related proxies—to capture the dynamics of energy usage throughout the manufacturing process. Several neural network architectures, including Long Short-Term Memory (LSTM), Gated Recurrent Unit (GRU), and Conv1D models, were trained and compared to predict short-term power demand with 10-min resolution. Among these, the GRU model achieved the highest predictive accuracy, with a best performance of RMSE = 2.18 kW, MAE = 0.49 kW, and SMAPE = 3.64% on the test set. The resulting forecasts support cost-efficient scheduling under time-of-use tariffs and provide valuable insights for infrastructure planning, capacity management, and sustainability optimization in energy-intensive industries.

## 1. Introduction

Accurate and reliable prediction of electricity demand is fundamental for optimizing energy consumption and achieving significant reductions in operational costs within industrial settings [1]. The ability to forecast future energy needs with precision enables proactive energy resource management, optimized procurement strategies, and streamlined production processes to minimize waste. Recognizing the limitations of traditional forecasting in capturing the complex dynamics of industrial energy usage, data-driven approaches, leveraging the sophisticated analytical capabilities of machine learning and deep learning techniques, have emerged as powerful and increasingly preferred tools for this critical task. Specifically, multi-sequence Long Short-Term Memory (LSTM)-based models have demonstrated effectiveness in short-term electric load forecasting by capturing temporal dependencies across multiple input sequences, offering a robust approach for predicting energy consumption patterns [2].

Recent scholarly investigations have extensively explored a wide range of methodologies that aim to precisely predict electricity demand on an hourly basis, often leveraging detailed consumption data available through time-of-use electricity billing systems [3,4]. These studies recognize the granular nature of such billing information as a valuable asset in discerning short-term load variations and improving the accuracy of forecasting for both residential and commercial consumers.

Building upon these detailed temporal analyses, the application of sophisticated data-driven techniques has expanded to encompass broader scales, demonstrating their effectiveness in forecasting aggregate electricity demand at the level of entire industries and sprawling urban areas [5]. This highlights the versatility and potential of these analytical approaches to provide valuable insights for energy management and planning across different levels of aggregation, from individual consumers to large-scale regional demands.

The application of advanced deep learning architectures has significantly enhanced the capabilities for forecasting complex energy demands, particularly in high-energy-consuming industrial sectors. Sophisticated models, such as the hybrid Convolutional Neural Network and Gated Recurrent Unit (CNN-GRU), have demonstrated a notable ability to predict multi-energy loads, effectively capturing the intricate interdependencies between various energy sources and their consumption patterns within these demanding industrial environments [6]. Furthermore, innovative deep learning models like the Bidirectional Gated Recurrent Unit (BI-GRU) encoder–decoder architecture have also proven to be highly effective in the broader task of electrical load forecasting, showcasing their capacity to learn and model the temporal dynamics inherent in electricity consumption time series data, leading to improved prediction accuracy across different industrial applications [7].

Beyond the realm of deep learning, traditional yet powerful statistical methodologies continue to play a vital role in electricity demand forecasting. Techniques employing Kalman filtering have shown promise in short-term load prediction by leveraging the inherent temporal dependencies within energy demand data and adapting to changing patterns. For instance, a study by Naderi et al. [8] explored a short-term load forecasting model based on an improved grey wolf optimizer and adaptive neuro-fuzzy inference system, utilizing a Kalman filter to enhance prediction accuracy. This demonstrates the continued relevance and potential of Kalman filtering in contributing to robust and accurate load forecasting models.

Complementing these model-based approaches, significant advancements in the utilization of standard load profiles are being achieved through the integration of cutting-edge data-driven techniques and the analysis of increasingly rich and recent datasets. By applying sophisticated analytical methods to these standardized consumption patterns, researchers are uncovering more nuanced insights and developing more accurate representations of typical energy usage, thereby enhancing the precision and applicability of these profiles for forecasting and energy management purposes across various sectors [9].

For large industrial consumers, characterized by complex and often highly variable energy consumption patterns, the application of targeted techniques such as feature preference and error correction has proven crucial in significantly enhancing the accuracy of load forecasting models. By carefully selecting the most relevant input features and implementing sophisticated mechanisms to identify and rectify prediction errors, these approaches can better capture the unique energy demand characteristics of these major users, leading to more reliable and actionable forecasts [10]. In parallel, the development of novel data-driven methodologies incorporating decomposition mechanisms offers another promising avenue for improving load forecasting across different temporal scales. These techniques work by breaking down the complex energy demand time series into more manageable components, each representing different underlying patterns or frequencies, which can then be modeled and forecasted individually before being aggregated to produce a final prediction, thereby improving the model’s ability to handle the multifaceted nature of electrical loads over varying time horizons [11].

To further enhance the accuracy and robustness of short-term electric load forecasting, researchers have increasingly explored the use of hybrid ensemble deep learning frameworks. These sophisticated approaches strategically combine the predictive strengths of multiple distinct deep learning models, often leveraging different architectures or training methodologies, to create a more powerful and reliable forecasting system that can outperform any single constituent model. By capitalizing on the diverse capabilities of individual networks, ensemble methods can better capture the multifaceted nature of electricity demand and improve prediction accuracy for immediate and near-future energy planning [12]. Complementing these broader methodological developments, the application of deep learning techniques to specific industrial consumer case studies has provided valuable real-world insights into the practical effectiveness and challenges of implementing these advanced forecasting models in diverse industrial settings. By examining the nuances of energy consumption within particular industrial facilities, these case studies contribute to a deeper understanding of the factors influencing load demand and help to refine deep learning models for improved performance in real-world applications [13].

In the pursuit of accurate short-term load forecasting for industrial customers, innovative hybrid models combining the strengths of different deep learning and machine learning architectures have emerged. One such promising approach involves the integration of Temporal Convolutional Networks (TCNs) with the gradient boosting framework LightGBM. TCNs excel at capturing temporal dependencies in sequential data, while LightGBM offers efficient and accurate gradient boosting capabilities, creating a powerful hybrid model for precise short-term predictions in industrial energy consumption [14]. Looking beyond short-term horizons, the challenge of long-term industrial electricity forecasting has also been addressed through advanced techniques. Deep learning models, capable of capturing intricate temporal patterns over extended periods, offer promising alternatives for this complex task. For example, Zhou et al. (2021) introduce the Informer model, an efficient transformer-based architecture specifically designed for long sequence time series forecasting, demonstrating its ability to effectively model long-range dependencies inherent in predicting future trends [15]. Such advanced deep learning techniques provide valuable tools for strategic energy planning and infrastructure development in industrial settings.

Further underscoring the versatility of Temporal Convolutional Networks (TCNs) in industrial energy forecasting, dedicated studies have demonstrated their effectiveness as standalone models for predicting industrial electrical energy consumption. Leveraging their ability to process sequential data efficiently and capture long-range dependencies, TCNs offer a robust deep learning approach for understanding and forecasting the energy demands of various industrial processes and facilities [16]. In addition to specific deep learning architectures, a broader range of machine learning-based algorithms has also been successfully applied to the task of short-term electric load forecasting in industrial plants. These diverse algorithms, encompassing methods from regression analysis to support vector machines and tree-based models, offer flexible and adaptable tools for capturing the complex relationships between various operational parameters and short-term fluctuations in electricity demand within industrial settings, providing valuable predictive capabilities for operational optimization and energy management [17].

Recognizing the complementary strengths of different forecasting paradigms, ongoing research actively explores the potential of bridging traditional statistical methods with the more recent advancements in deep learning techniques for electricity load forecasting. These investigations aim to leverage the interpretability and robustness of statistical models alongside the pattern recognition and complex feature extraction capabilities of deep learning, potentially leading to hybrid approaches that offer enhanced accuracy and a deeper understanding of the underlying drivers of energy demand [18]. Furthermore, the increasing availability of high-frequency energy consumption data and the demand for immediate operational insights have spurred the development of deep learning approaches specifically designed for real-time electricity load forecasting. These models are engineered to process and analyze streaming data with minimal latency, providing timely and dynamic predictions that can support immediate decision-making in energy management systems, grid operations, and demand response initiatives, enabling more agile and responsive energy management strategies [19].

In recent years, short-term electricity load forecasting has become a fundamental tool for energy planning and smart grid operation. Various data-driven approaches have demonstrated effectiveness in accurately predicting day-ahead demand. For instance, Inteha et al. [20] employed machine learning and statistical techniques for day-ahead load forecasting, highlighting the adaptability of data-driven models to dynamic consumption patterns. In the Colombian context, García-Guiliany et al. [21] implemented multiple linear regression models to project electricity demand, showing that classical statistical approaches remain valuable for low-complexity forecasting tasks. More recently, Toledo-Cortés et al. [22] provided a detailed characterization of electricity demand based on real consumption data from Colombia, offering a robust foundation for the development of predictive models. Additionally, Moreno-Chaparro et al. [23] combined wavelet analysis with nonlinear autoregressive models to forecast monthly electricity demand in Colombia, capturing multi-scale temporal variability and improving predictive accuracy. These efforts demonstrate the importance of integrating advanced data analysis and statistical modeling techniques to strengthen forecasting capabilities in emerging power systems.

Recent studies have highlighted the growing importance of machine learning-driven energy-saving strategies for improving energy management in industrial systems. For instance, Morgoeva et al. [24] demonstrated how supervised learning models can be effectively used to forecast industrial electricity consumption and support tactical decision making in manufacturing enterprises, enabling cost reduction and reserve capacity optimization. Complementarily, a recent contribution by Van et al. [25] addressed energy consumption minimization in cyber–physical and networked robotic systems, emphasizing the interdisciplinary integration of machine learning, communication networks, and energy-aware control. These studies confirm that accurate data-driven forecasting is a key enabler for both tactical and strategic energy management, strengthening the economic efficiency, operational flexibility, and sustainability of modern industrial infrastructures.

### 1.1. Classical Modeling and Complementarity with Data-Driven Forecasting

Recent doctoral work has emphasized that robust forecasting frameworks for local energy systems must balance both predictive accuracy and practical value, often combining state-of-the-art deep learning architectures—such as N-BEATS—with classical baselines like ARIMA, XGBoost, and Prophet, while embedding these forecasts into Model Predictive Control (MPC) frameworks to optimize cost and performance [26]. Inspired by this rationale, our study adapts these principles to an industrial quicklime production context, where electricity demand is heavily influenced by thermally intensive unit operations. In this setting, reliable short-term forecasts generated by compact GRU-based models support tariff-aware scheduling, constraint handling, and scenario-based capacity planning.

Beyond purely statistical or deep learning models, the integration of first-principles and grey-box approaches remains essential for capturing the underlying physical dynamics of processes such as grinding, calcination, and material conveying. These models enable scenario-based reasoning and simulation via frameworks like the Specific Load by Representative Stage (SLRS), which decomposes industrial systems into representative energy-consuming stages and aligns well with operational planning. Our contribution is designed to complement this body of work: the proposed GRU forecaster, trained on historical electrical and calendar features, serves as a high-resolution, feed-forward input to hybrid MPC systems. In doing so, it functions as a boundary condition, calibration signal, or supervisory reference, enhancing the performance and robustness of advanced control strategies. This integration bridges the gap between data-driven forecasting and physics-based control architectures, aligning with recent efforts to build more flexible, explainable, and robust industrial energy management systems [27,28].

### 1.2. Forecasting as an Enabler for Advanced Energy Management

Recent advancements in model predictive control (MPC) and cyber–physical energy systems increasingly emphasize resilience against uncertainties, disturbances, and cyber-security threats. For example, Ma et al. [29] proposed an event-triggered multi-kernel learning-based stochastic MPC for building climate optimization, while He et al. [30,31] addressed resilience against false data injection and deception attacks in nonlinear control systems. These studies illustrate a broader trend toward integrating estimation, learning, and control in energy systems.

In this context, accurate short-term load forecasting plays a fundamental enabling role, as it provides the predictive input required by advanced supervisory and optimization layers. The present work focuses specifically on the development and validation of a high-resolution forecasting model for an energy-intensive quicklime production process. Rather than implementing MPC or cyber-security mechanisms, our contribution is positioned at the forecasting layer, supplying reliable demand predictions that can support future integration with tariff-aware scheduling, optimization, and secure control architectures [32,33].

### 1.3. Overview of the Study and Paper Organization

This study presents the development of a model for predicting electricity load profiles using energy consumption variables. We compare LSTM, GRU, and conv1D forecasters. The low RMSE values observed demonstrated the effectiveness of the method. The article is organized as follows: Section 2 reviews the literature on load profile prediction, load simulators, and energy consumption time series forecasting. Section 3 describes the materials and methods used, including an overview of the quicklime production process and the machinery at SUMININCO LTDA, along with their power consumption. This section also details the data preprocessing steps, and the methodology for time series forecasting using GRU, outlining each stage. Section 4 presents the results, showing the predictions from the GRU model against actual data, calculating RMSE on the test set, and conducting parameter tuning. Finally, Section 5 concludes with the main findings of the study.

## 2. Literature Review

In their review, Iqbal et al. [34] examined a range of Demand Side Management (DSM) strategies aimed at reducing residential energy consumption and improving grid stability in smart grid environments. The authors categorized these strategies into energy conservation and efficiency, demand response (DR), energy optimization and scheduling, distributed generation, and energy storage, with each analyzed based on its specific application, such as peak shaving, valley filling, and load shifting. They also evaluated the implementation of DSM through soft computing techniques like Fuzzy Logic (FL), Artificial Neural Networks (ANN), and Evolutionary Computation (EC), as well as optimization methods including Game Theory and Mixed-Integer Linear Programming (MILP). Results showed that DSM strategies contribute significantly to energy conservation, economic efficiency, and balancing demand and supply, which are vital for smart grid stability. Soft computing methods, particularly ANN and FL, were found to be highly effective for DSM in residential applications, while optimization techniques like MILP and Game Theory proved advantageous for load scheduling and cost reduction. The review highlighted the need for high-resolution data in DSM modeling but noted obstacles such as data privacy and limited public datasets. The authors recommended further research into real-time DSM applications, renewable energy integration, and user engagement approaches, emphasizing the importance of advanced, privacy-focused data solutions to improve DSM in smart grids.

Recent advances in technology, such as smart meters and advanced data analytics, have made it easier to perform energy disaggregation. These tools can help identify patterns and provide insights into energy consumption, ultimately contributing to energy savings and improved efficiency [35]. Over the past few years, advances in mathematical theory and modern computing have led to ongoing improvements in electricity consumption forecasting models. These models include the economic model, the comprehensive analysis model, and the classification-based forecasting model [36].

In their comprehensive review, Proedrou [37] examined residential electricity load profile models, categorizing them to identify common features and challenges. The authors conducted an extensive literature search and proposed a new classification system based on methodology (bottom-up, top-down, and hybrid), sampling rate (low, middle, and high resolution), primary application (demand-side management, energy systems planning/control, and load profiling), and statistical techniques (Markov chains, probabilistic methods, and Monte Carlo simulations). This classification enabled a detailed analysis of model applications, ranging from individual appliance usage to regional residential consumption. The review highlighted that most models use bottom-up methods for detailed load profiles, suitable for energy optimization and demand response simulations, but noted a scarcity of high-resolution models due to privacy and data collection issues. Few models capture load profile variability from factors like remote work or electric vehicle use. Proedrou emphasized the importance of middle- and high-resolution models for demand-side management and load shifting, especially with increased renewable integration, and recommended solutions such as anonymized data sharing and crowd-sourced datasets to improve data access and model accuracy.

In their study, González López et al. [38] developed a smart residential load simulator (SRLS) using MATLAB-Simulink to model energy consumption for various residential appliances and renewable energy sources within smart grid environments. The SRLS includes user-friendly graphical interfaces for simulating appliances such as air conditioners, water heaters, stoves, dishwashers, washing machines, lighting, and pool pumps, with models validated against real-world measurements. It also allows for the integration of local energy generation from photovoltaic and wind sources, as well as battery storage, providing a holistic view of residential energy dynamics. Key inputs such as ambient temperature, time-of-use tariffs, and user schedules enable realistic simulations of household energy consumption. The SRLS generates appliance-specific load profiles that can be aggregated to assess their impact on peak demand and overall energy costs. Validation results showed strong alignment with measured data, confirming the SRLS’s accuracy in modeling daily load profiles. Additionally, the SRLS was effective in simulating smart load management scenarios, demonstrating its potential for energy management, optimization, and smart grid applications by supporting load shifts and peak reduction in response to demand signals.

The work of Sandhaas et al. [39] proposed a methodology to create synthetic load profiles for different industrial sectors, addressing the demand for high-resolution electricity consumption data in energy system modeling. The approach included four steps: generating daily normalized load profiles for eight end-use applications (e.g., space heating, process heat, mechanical drives), clustering industry types based on the German classification system (WZ 2008) with a focus on food, metal products, and machinery, refining profiles by categorizing mechanical drive processes as continuous or discontinuous, and applying a synthetic fluctuation rate to introduce realistic load variability. Additionally, the regional distribution of industry types was considered to adapt the profiles to various geographic areas. Results showed that these synthetic profiles closely matched real industrial demand, particularly for three-shift operations, achieving low Root Mean Square Error (RMSE) values when validated against actual load data. This methodology, especially effective for three-shift industries, also supports demand-side management and energy flexibility modeling, proving accurate enough for integration into high-resolution models like MyPyPSA-Ger.

Mocanu et al. [40] explored two deep learning methods—Conditional Restricted Boltzmann Machine (CRBM) and Factored Conditional Restricted Boltzmann Machine (FCRBM)—for predicting time series energy consumption using the “Individual household electric power consumption” dataset, containing minute-resolution data over four years, split into three years for training and one for testing. To handle approximately 1.25% missing data, they filled gaps with the average power consumption at corresponding times from other years. The authors benchmarked CRBM and FCRBM against other models, including Artificial Neural Networks (ANN), Support Vector Machines (SVM), and Recurrent Neural Networks (RNN). The CRBM used historical data to forecast short- and long-term consumption based on temporal dependencies, while the FCRBM, an enhanced CRBM variant, introduced a “style” layer to capture unique consumption patterns and applied multiplicative interactions to handle complex dependencies. Both models were trained with Contrastive Divergence and utilized recursive multi-step predictions to extend forecast horizons. The results showed that FCRBM outperformed all models, consistently achieving lower RMSE values across various prediction intervals, especially in high-variability scenarios like one-day predictions. While CRBM surpassed baseline models, its performance was less robust over longer forecast periods than FCRBM, which demonstrated resilience and accuracy across all intervals. The study concluded that FCRBM’s architecture is well-suited for real-time applications in smart grids and building energy management, particularly due to its strength in capturing non-linear time series patterns.

In their study, Marino et al. [41] employed two variations of Long Short-Term Memory (LSTM) architectures—standard LSTM and Sequence to Sequence (S2S) LSTM—to forecast building-level energy loads. Using the “Individual household electric power consumption” dataset, which includes electricity data with one-minute resolution (later averaged to one-hour for comparison), they trained the models on three years of data and reserved the fourth year for testing. The standard LSTM used active power measurements from previous time steps and temporal features (day of the week, hour of the day), with Backpropagation Through Time (BPTT) and the ADAM optimizer for training. In contrast, the S2S architecture involved two LSTM networks—an encoder and a decoder—allowing it to handle variable input lengths and produce flexible multi-step forecasts. Performance was evaluated based on RMSE across datasets, with Dropout regularization applied to prevent overfitting and various configurations tested for optimal performance. Results showed that while the standard LSTM performed well for single-step predictions on hourly data, it struggled with multi-step forecasts on one-minute data due to naive outputs. The S2S architecture, however, achieved superior accuracy, maintaining low RMSE values across both time resolutions, and provided robust forecasts, proving comparable to the advanced Factored Conditional Restricted Boltzmann Machines (FCRBM) used in prior research.

An unsupervised approach for time series forecasting was developed by Franceschi et al. [42] generating versatile representations of multivariate time series using a convolutional encoder with dilated causal convolutions, which captures temporal dependencies and scales to varying time series lengths. A unique triplet loss function, utilizing time-based negative sampling, trained the encoder without supervision by selecting positive and negative samples based on temporal relevance, ensuring that unrelated sequences had distinct representations. The encoder, optimized with Adam, produced fixed-length vector representations that generalized well across various time series tasks. Through experiments on the UCR and UEA time series datasets, Franceschi et al. showed that their method outperformed other unsupervised techniques, achieving near state-of-the-art accuracy for univariate data and competitive results for multivariate data. The method also demonstrated scalability on the long time series dataset “Individual Household Electric Power Consumption,” proving its capacity to generate efficient representations at different temporal resolutions. These findings highlight the method’s potential for diverse applications in unsupervised time series analysis, including clustering, anomaly detection, and regression.

Gasparin et al. [43] conducted a comprehensive assessment of deep learning models for electric load forecasting by evaluating multiple architectures, including Feed-Forward Neural Networks (FNNs), Recurrent Neural Networks (RNNs), Long Short-Term Memory (LSTM) models, Gated Recurrent Units (GRU), and Temporal Convolutional Networks (TCN). Using datasets with varying temporal resolutions, the authors applied each model to predict load consumption for different time horizons, testing both individual and aggregated demand scenarios. They also implemented several multi-step forecasting strategies—recursive, direct, and sequence-to-sequence methods—analyzing their influence on accuracy and efficiency. Model performance was evaluated based on metrics such as RMSE, Mean Absolute Error (MAE), and R-squared, revealing that TCNs and GRU models generally outperformed others, particularly in capturing short-term load patterns. Gasparin et al. concluded that deep learning approaches, specifically TCNs and GRUs, are highly effective for load forecasting tasks, with TCNs providing robust predictions for aggregated loads and demonstrating potential for smart grid applications due to their parallel processing capabilities.

Recent studies have increasingly addressed electricity demand forecasting in industrial and manufacturing environments, where energy consumption is driven by tightly coupled production processes, machine operation cycles, and material flows. Walther and Weigold [44] analyzed machine-learning-based modeling of electrical energy consumption in manufacturing systems, highlighting the strong dependency between production scheduling, process parameters, and electrical demand. Similarly, Verwiebe et al. [45] conducted a systematic literature review on industrial energy demand modeling, emphasizing the importance of high-resolution measurements and process-level data to capture the nonlinear behavior of industrial loads.

More application-oriented works have demonstrated the integration of artificial intelligence into smart manufacturing energy management. Hsu et al. [46] proposed an AI-driven framework for demand prediction and optimization in smart grids applied to industrial facilities, showing significant improvements in operational efficiency and energy utilization. In addition, Rojek et al. [47] developed a green energy management approach for manufacturing systems based on short-term demand prediction using artificial intelligence, reinforcing the role of forecasting as a key enabler for sustainability-driven industrial decision-making.

Despite these advances, most existing studies focus on general manufacturing environments, whereas energy-intensive thermo-mechanical processes such as quicklime production remain largely underexplored. Unlike generic industrial plants, quicklime manufacturing exhibits highly structured operating schedules, strong coupling between mechanical drives and thermal calcination stages, and strict production constraints. The present study addresses this gap by developing a data-driven forecasting framework specifically tailored to a real quicklime production facility, using high-resolution electrical measurements, operational indicators, and process-related variables to enable tariff-aware scheduling and industrial energy optimization.

### Research Gaps and Motivations

Despite the significant progress achieved in short-term electricity load forecasting through deep learning and hybrid data-driven approaches, several relevant research gaps remain open.

1 First, most existing works focus on residential, commercial, or aggregated urban loads, while high-resolution forecasting studies addressing energy-intensive industrial processes—such as quicklime manufacturing—remain scarce. These processes are characterized by strong operational discontinuities, thermally driven unit operations, and highly non-linear demand patterns that are insufficiently represented in current forecasting benchmarks.
2 Second, although advanced models such as LSTM, GRU, TCN, CNN-GRU, and transformer-based architectures have been widely explored, limited attention has been given to the combined and systematic integration of temporal, operational, and autoregressive features under real industrial operating conditions. In particular, the joint influence of shift indicators, production-related proxies, and short-term load memory has not been sufficiently quantified in heavy-process industries.
3 Third, while numerous studies report forecasting accuracy improvements, fewer works explicitly connect short-term forecasting models with their downstream industrial implications, such as tariff-aware scheduling, infrastructure planning, and capacity management in energy-intensive plants located in emerging economies.

Motivated by these gaps, this study proposes a high-resolution GRU-based forecasting framework using one year of real industrial electricity consumption data from a quicklime production plant in Colombia. The proposed approach integrates temporal, operational, and load-history features, systematically compares deep learning architectures (LSTM, GRU, and Conv1D), and establishes a direct link between forecasting accuracy and tariff-aware operational decision making. In doing so, the study aims to contribute both methodologically and practically to the development of reliable, scalable, and industry-oriented forecasting tools for energy-intensive manufacturing systems.

## 3. Materials and Methods

### 3.1. Materials

SUMININCO LTDA is a company located in Nobsa, Boyacá, specialized in the production and marketing of steel lime, industrial lime and lime for agricultural use (Calcium Hydroxide). For more information, the reader can consult the SUMININCO LTDA website at the following link: https://sumininco.com/index.html (accessed on 3 October 2025). In order to identify the different processes involved in the manufacture of lime, the production process of this is detailed below:

Quicklime, also known as calcium oxide (CaO), is produced by a process called calcination, which involves heating calcium carbonate (CaCO_3_) to high temperatures. This process is carried out in special furnaces known as calcining kilns. The following are the general stages of the LIME production process:

1 Extraction of limestone: Calcium carbonate is commonly found in the form of limestone. This limestone is extracted from quarries using mining techniques.
2 Crushing and grinding: Limestone is crushed and ground into smaller particles to increase the reaction surface during the calcining process.
3 Feeding to the kiln: Crushed limestone is fed into the calcining kiln. The kiln operates at high temperatures typical of industrial calcination, usually around 900–1000 °C (1652–1832 °F), which are well within the design range for CaCO_3_ → CaO conversion.
4 Calcination: Inside the kiln, calcium carbonate is thermally decomposed into calcium oxide (quicklime) and carbon dioxide (CO_2_), according to the following chemical reaction:
  $${\mathrm{CaCO}}_{3}\left(s\right)\to \mathrm{CaO}(s)+{\mathrm{CO}}_{2}\left(g\right)$$
5 Collection and cooling: The resulting calcium oxide is collected at the bottom of the kiln and transported for cooling. During this process, the quicklime is cooled to prevent unwanted reactions and to facilitate its handling and storage.
6 Storage and distribution: Once cooled, the quicklime is stored in suitable containers and distributed for use in a variety of industrial and commercial applications, including steel production, cement manufacturing, water treatment, gas purification, and many other uses.

Specifically, the lime production process at SUMININCO Ltda. begins in the yard where the material to be processed arrives. A Hopper 1 (20 HP motor) is then used to control the flow of the material that will enter the crusher (30 HP motor). Then, the crushed material is transported without screening by a belt 1 (3 HP gear motor). The next step in the process is the screening of the material, which is responsible for allowing a specific size of material to pass through. The sieve has a 25 HP motor. The material at the exit of the sieve is transported by a belt 2 (3 HP motor) to a bucket elevator (3 HP motor). Then there is a belt 3 (3 HP motor) that will take the material to the entrance of the oven. Specifically, the continuous vertical oven has a tank where the material is calcined. This oven works with coke. Coke is a solid product obtained from the distillation of bituminous coal at high temperatures in the absence of air. It is important to note that the amount of coke used in the furnace is a fundamental piece in the cost equation of production at SUMININCO Ltda. There is a 4-belt (2 HP motor) in a sky-type elevator that takes the material to the furnace. The furnace has an induced draft system of air supplied by fans. This system is not used permanently. At the furnace exit, there is a 2 HP motor. After the material at the furnace exit (Lime), there is a toothed roller mill with an 11 HP motor.

It is important to note that electrical measurements in this study are obtained from a single main energy meter installed at the plant level. This meter records the aggregated active power consumption of the entire production line, including all motors associated with crushing, conveying, sieving, calcination, ventilation, and milling. Therefore, individual motor-level electrical measurements are not available. The relationship between specific process stages and electrical demand is inferred qualitatively based on the known operating sequence of the equipment and the production schedule.

### 3.2. Data Acquisition and Quality Assessment

The experimental dataset used in this study was collected from an industrial energy monitoring system installed at the main electrical distribution board of the production facility. Active power consumption was measured using an ACTARIS/ITRON AC6000 (Itron, Inc., Liberty Lake, WA, USA) smart energy meter, accuracy class 0.5 S, rated at 5(10) A and 57–240/415 V ±20%. The meter operates in bidirectional mode and supports both two- and three-element self-powered configurations. It is equipped with one RS-232 communication port and one IEC 61107 optical port, and provides a dual load curve with 8 + 8 internal memory channels. The meter was calibrated by a laboratory accredited by the Superintendencia de Industria y Comercio (SIC) in Colombia, ensuring traceability and measurement reliability.

Remote data transmission was carried out using a ROBUSTEL M1201 4G industrial communication modem (Robustel Technologies Co., Ltd., Guangzhou, China) with SIM card, enabling secure encrypted data communication. The modem supports serial data transmission through RS-232 and RS-485 ports and was equipped with an external antenna and a dedicated power supply unit, ensuring stable and continuous operation under industrial conditions.

The data were recorded at a fixed sampling interval of 10 min, resulting in high-resolution multivariate time-series data suitable for short-term energy forecasting. The complete dataset spans several consecutive weeks of operation, covering both production and non-production periods.

Regarding data completeness, the percentage of missing samples was below 1.5% of the total dataset. Missing values were handled using linear interpolation for isolated gaps shorter than three consecutive samples, while longer gaps were discarded from the analysis to avoid the introduction of bias.

Anomaly detection was performed using a two-stage procedure. First, a threshold-based filter was applied to remove physically impossible values (negative power and spikes exceeding the rated electrical capacity of the installation). Second, a rolling statistical filter based on the median absolute deviation (MAD) was employed to identify and remove outliers associated with sensor noise or communication errors.

All signals were subsequently normalized using Min–Max scaling prior to model training. In addition, production shift indicators and process-related variables were temporally synchronized with the power measurements to ensure full temporal consistency across all input features.

### 3.3. Data Set Description

The methodology uses the load profile of the quicklime production process over a full year, taking into account working hours and variations in motor usage. It defines key parameters such as the number of days per year, hours per day, and minutes per hour, as well as the power ratings of the motors, which are converted from horsepower to watts. The load profile operates exclusively during business hours (8:00 a.m. to 4:00 p.m.) on weekdays, reflecting the plant’s real operating schedule. The resulting data are organized into a structured table and saved as a CSV file for further analysis, enabling the detailed examination of load behavior under different operational conditions. The data was captured every 10 min from 1 January 2024 to 31 December 2024. The dataset has 52,704 rows.

**Figure 1:**
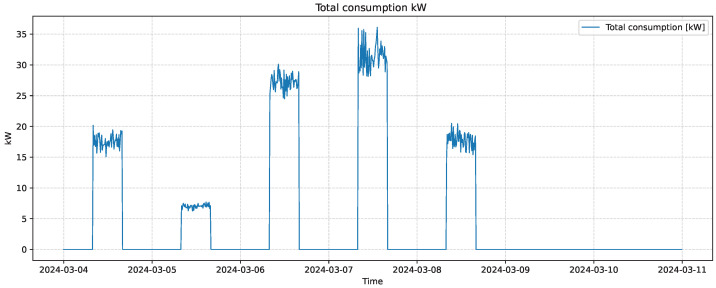
Weekly load profile of the quicklime production facility showing total electrical consumption (kW).

Figure 1 illustrates the total electrical power consumption of the quicklime production facility over one representative week. The load profile exhibits a distinct operational pattern, with energy usage concentrated exclusively during working hours (08:00–16:00) from Monday to Friday, and zero consumption during non-working hours and weekends. The variations in power demand within each active period reflect the sequential operation of different production stages—such as crushing, sieving, and grinding—each associated with distinct motor loads. The observed peaks correspond to higher energy requirements during material processing, while shorter fluctuations are attributed to normal variability in motor utilization and process transitions. This characteristic weekly pattern confirms the strong correlation between production scheduling and electrical load behavior in the plant.

It is important to note that the industrial facility analyzed in this study is located in Colombia, which is characterized by a tropical climate where the classical four-season annual climatic pattern is not present. Consequently, long-term climatic seasonality associated with temperate regions is not a governing factor of the production process. Instead, the dominant seasonal behavior of the energy demand is driven by operational schedules, production shifts, and recurrent weekly demand cycles. These forms of operational seasonality are explicitly captured by the temporal and operational features included in the GRU model.

The variable coke_kg, which represents the mass of coke used during the calcination stage, is obtained from the plant’s daily operational records. In SUMININCO LTDA, the coke is manually weighed during the furnace feeding process as part of routine production control. Since these records are available at daily resolution, the measured coke mass is synchronized with the 10-min electrical consumption dataset by applying constant interpolation over each corresponding operating shift. This procedure allows the coke consumption indicator to be consistently integrated as an operational feature in the forecasting model.

### 3.4. Feature Engineering

To encode daily periodicity, the hour-of-day h∈{0,…,23} is mapped into two cyclic features,

(1) $$\mathrm{Hour}\_\sin=\sin\;\left(\frac{2\pi h}{24}\right),\;\mathrm{Hour}\_\cos=\cos\;\left(\frac{2\pi h}{24}\right),$$

which avoid discontinuities between 23:00 and 00:00. In addition, autoregressive information is injected through rolling means of the active power $P_{t}$ computed using overlapping sliding windows of 1 h and 2 h:

(2) $$\mathrm{roll}\_1h\left(t\right)=\frac{1}{6}\sum_{k=0}^{5}P_{t-k},\;\mathrm{roll}\_2h\left(t\right)=\frac{1}{12}\sum_{k=0}^{11}P_{t-k}.$$

The predictive feature vector includes operational indicators (is_shift, coke_kg), temporal variables (Hour_sin, Hour_cos, Day, Month), and autoregressive inputs (load_now, roll_1h, roll_2h).

The target is defined for one-step-ahead forecasting as

(3) $$y_{t+1}=P_{t+1},$$

i.e., the active power 10 min after time *t*. All feature construction strictly uses information available at or before time *t* to prevent leakage. The input and output variables used in the forecasting model are summarized in Table 1.

### 3.5. Normalization and Temporal Sequencing

All features are scaled to [0,1] using Min–Max normalization. To avoid bias from prolonged off-shift periods (where $P_{t}\approx 0$), the target scaler is fitted *only* on training samples belonging to active shifts. Given the lookback L=48, training examples are built as overlapping sequences

(4) $$X_{i}=\left[x_{i-L},\ldots ,x_{i-1}\right],\;y_{i}=P_{i},$$

where $x_{t}$ is the feature vector at time *t*.

During training, samples within production shifts are emphasized via a weighting factor α=3.0, while off-shift samples receive unit weight. This improves fidelity in the operating regime where accurate prediction is most critical.

### 3.6. Model Architecture and Training

This section outlines the forecasting pipeline adopted to predict short-term electric power demand in a quicklime manufacturing plant. The proposed approach relies on a Gated Recurrent Unit (GRU) neural network trained on time-series data with a 10-min sampling resolution. An 8-h temporal window (48 time steps) is used as input context, and the dataset is chronologically divided into 80% for training and 20% for testing in order to preserve temporal dependencies.

During the hyperparameter tuning stage, a single-layer GRU architecture was adopted to evaluate the effect of the number of recurrent units (50, 100, and 150), activation functions, and optimizers in a controlled manner. After this tuning phase, the final forecasting architecture was defined as a two-layer GRU network with 64 and 32 hidden units, respectively, which was then used consistently in all subsequent comparative and validation experiments. GRUs were selected for their ability to model long temporal dependencies with fewer parameters than LSTMs. The model is optimized with Adam (learning rate $10^{-3}$) and trained using the Huber loss, which is robust to occasional outliers compared to mean squared error. Early stopping with a patience of five epochs restores the best weights according to validation loss. The batch size is 64 and the maximum number of epochs is five (sufficient for convergence given the data regularity).

The selection of the number of GRU layers and hidden units was guided by a systematic hyperparameter sensitivity analysis, whose quantitative results are reported in Section 4. The number of units was varied in the range of 50 to 150, combined with different activation functions (ReLU and tanh) and optimization algorithms (Adam and RMSprop). The final configuration was chosen based on the best compromise between predictive accuracy and model complexity, prioritizing low RMSE and MAE values while avoiding excessive model depth that could lead to overfitting and unnecessary computational cost.

In addition, a two-layer GRU topology with progressively decreasing hidden units (64 and 32 units, respectively) was adopted to enable hierarchical temporal feature extraction while maintaining compact model size. The first GRU layer captures higher-level temporal patterns, whereas the second layer refines short-term dependencies, resulting in improved generalization without increasing overfitting risk.

### 3.7. Evaluation Protocol

Performance is reported on the held-out test set using root mean square error (RMSE), mean absolute error (MAE), and symmetric mean absolute percentage error (SMAPE). SMAPE is defined as

(5) $$\mathrm{SMAPE}\left(\hat{y},y\right)=\frac{100}{N}\sum_{t=1}^{N}\frac{\left|{\hat{y}}_{t}-y_{t}\right|}{\left(|{\hat{y}}_{t}\left|+\right|y_{t}|\right)/2+\varepsilon },$$

with a small ε to avoid division by zero. For physical consistency, negative predictions are clipped to zero, and a post-processing mask sets predictions to zero outside production shifts.

All SMAPE values reported in this study are computed using the same formulation and the same final post-processing strategy, including clipping of negative predictions and masking outside production shifts. Unlike the preliminary tuning experiments, all comparative and validation results are reported after enforcing zero load during non-operational periods.

For physical consistency, negative predictions are clipped to zero according to

(6) $${\hat{y}}_{t}=\max\left({\hat{y}}_{t},0\right),$$

and predictions outside production shifts are masked using the binary operational indicator is_shift, such that the final corrected forecast is given by

(7) $${\hat{y}}_{t}^{\mathrm{final}}={\hat{y}}_{t}\cdot \mathrm{is}\_{\mathrm{shift}}_{t}.$$

This post-processing procedure enforces non-negative power estimates and prevents spurious load predictions during non-operational periods.

#### Reproducibility Notes

Key hyperparameters are: lookback L=48 (8 h), train/test split 80/20 in chronological order, batch size 64, Adam learning rate $10^{-3}$, shift weight α=3.0. Feature lists, scaling strategy (target scaler fitted on in-shift samples), and the exact sequencing procedure are provided above to enable faithful reproduction.

### 3.8. Time Series Forecasting

Unlike classification and regression problems, time series problems add the complexity of order or temporal dependence between observations. This can be difficult as specialized data handling is required when fitting and evaluating models. This temporal structure can also aid in modeling, providing additional structure such as trends and seasonality that can be leveraged to improve model skill. Traditionally, time series forecasting has been dominated by linear methods such as the autoregressive integrated moving average model (ARIMA) because they are effective in many problems. However, classical methods such as ARIMA suffer from some limitations, such as:

- They focus on complete data: missing or corrupted data is usually not supported.
- They focus on linear relationships: assuming that a linear relationship excludes more complex joint distributions.
- They focus on fixed temporal dependence: the relationship between observations at different times, and in turn the number of lag observations provided as input, must be diagnosed and specified.
- They focus on univariate data: Many real-world problems have multiple input variables.
- They focus on single-step forecasts: Many real-world problems require forecasts with a long time horizon.

In contrast to linear methods such as ARIMA, machine learning methods are effective on more complex time series prediction problems with multiple input variables, complex nonlinear relationships, and missing data. The data set obtained for the electric load profile of the company Sumininco LTDA has properties of a time series where there are data that are repeated sequentially in time. For example, there is load profile data for 5 days a week worked (Monday to Friday) with a workday that starts at 8:00 a.m. and ends at 4:00 p.m.

#### 3.8.1. Deep Learning Recurrent Neural Networks

Deep learning neural networks can automatically learn arbitrary complex input-to-output mappings and support multiple input and output variables. Convolutional neural networks (CNNs), recurrent neural networks (RNNs), and a combination of both are used to discover subtle relationships and structures in high-dimensional data and in sequences with temporal context. These features are well suited for time series forecasting, particularly in problems with complex non-linear dependencies, multi-valued inputs and for producing multi-step forecasts. They can be applied to many real-world contexts such as solving complex classification, text categorization, computer vision, image processing and speech recognition problems.

#### 3.8.2. Recurrent Neural Networks Using LSTM Cells

Long Short-Term Memory-based neural networks (LSTMs) are an advanced type of recurrent neural networks (RNNs) specifically designed to learn long-term dependencies in data sequences. Unlike traditional RNNs, LSTMs feature a structure of memory cells that allow key information to be retained over time, which helps them overcome the gradient vanishing problem. These cells are controlled by three types of gates: the input gate, the output gate, and the forget gate, which regulate the flow of information in and out of the cell. Thanks to this architecture, LSTMs are highly effective in tasks that require learning patterns from long sequences, such as natural language processing, time series prediction, and machine translation.

#### 3.8.3. Recurrent Neural Networks Using GRU Cells

Gated Recurrent Unit (GRU) networks are a variant of recurrent neural networks (RNNs) developed to address the limitations of traditional RNNs in learning long-term dependencies, similar to LSTMs but with a simplified architecture. GRUs combine the memory and gating mechanisms into two gates: the update gate and the reset gate. The update gate controls how much of the previous memory is retained, while the reset gate determines how to combine the new input with the past memory. This design reduces computational complexity compared to LSTM networks while still mitigating the vanishing gradient problem. GRUs are particularly effective in time series prediction tasks, offering competitive performance with fewer parameters, which makes them suitable for applications requiring efficient training and inference, such as energy forecasting and real-time monitoring.

#### 3.8.4. Gated Recurrent Unit (GRU): Mathematical Formulation and Integration with the Data Processing Pipeline

The Gated Recurrent Unit (GRU) is a recurrent neural network architecture designed to efficiently capture temporal dependencies in sequential data while mitigating the vanishing gradient problem commonly encountered in traditional recurrent neural networks (RNNs) [48,49]. In this study, the GRU constitutes the core predictive model used for energy demand forecasting. For completeness and clarity, its full mathematical formulation is detailed below.

Let $x_{t}\in R^{n}$ denote the input feature vector at time *t*, and let $h_{t}\in R^{m}$ represent the hidden state of the GRU. The internal dynamics of the GRU are governed by two gating mechanisms: the update gate $z_{t}$ and the reset gate $r_{t}$, which are computed as

(8) $$z_{t}=\sigma \left(W_{z}x_{t}+U_{z}h_{t-1}+b_{z}\right),$$

(9) $$r_{t}=\sigma \left(W_{r}x_{t}+U_{r}h_{t-1}+b_{r}\right),$$

where σ(·) denotes the logistic sigmoid activation function, $W_{z},W_{r}\in R^{m\times n}$ are the input weight matrices, $U_{z},U_{r}\in R^{m\times m}$ are the recurrent weight matrices, and $b_{z},b_{r}\in R^{m}$ are bias vectors.

The reset gate $r_{t}$ regulates how much of the past hidden information is retained when computing the candidate hidden state ${\tilde{h}}_{t}$, which is defined as

(10) $${\tilde{h}}_{t}=\tanh\left(W_{h}x_{t}+U_{h}\left(r_{t}⊙h_{t-1}\right)+b_{h}\right),$$

where ⊙ denotes the Hadamard (element-wise) product, $W_{h}\in R^{m\times n}$ and $U_{h}\in R^{m\times m}$ are weight matrices, and $b_{h}\in R^{m}$ is the bias vector associated with the candidate activation.

Finally, the hidden state $h_{t}$ is updated as a convex combination of the previous hidden state $h_{t-1}$ and the candidate state ${\tilde{h}}_{t}$, controlled by the update gate $z_{t}$:

(11) $$h_{t}=\left(1-z_{t}\right)⊙h_{t-1}+z_{t}⊙{\tilde{h}}_{t}.$$

**Figure 2:**
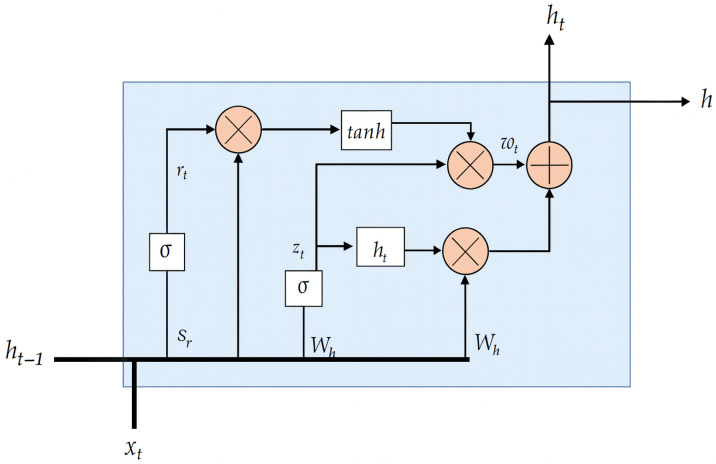
Schematic diagram of a Gated Recurrent Unit (GRU) cell. The input at time step *t* is represented by the feature vector $x_{t}$, while $h_{t-1}$ denotes the previous hidden state. The reset gate $r_{t}$ controls the contribution of past information to the candidate hidden state ${\tilde{h}}_{t}$, which is computed through a hyperbolic tangent activation function. The update gate $z_{t}$ regulates the interpolation between the previous hidden state $h_{t-1}$ and the candidate state ${\tilde{h}}_{t}$, producing the new hidden state $h_{t}$, which also generates the model output $y_{t}$ at the current time step. Adapted from [50].

This formulation highlights the dual role of the update gate in determining both the memory retention and the incorporation of new temporal information. Through this adaptive mechanism, the GRU is able to dynamically balance short-term fluctuations and long-term temporal dependencies in the energy consumption time series. Figure 2 shows the schematic diagram of a Gated Recurrent Unit (GRU) cell.

#### 3.8.5. Connection Between Feature Engineering and the GRU Internal Mechanisms

The components described in Section 3.3, Section 3.4, Section 3.5 and Section 3.6, including signal normalization, temporal windowing, lag feature construction, and exogenous variable integration, directly determine the structure of the input vector $x_{t}$ at each time step. Specifically, the engineered features define the dimensionality *n* of $x_{t}$ and strongly influence the activation patterns of the update and reset gates.

The reset gate $r_{t}$ is particularly sensitive to abrupt variations and transient patterns introduced by short-term features, enabling the GRU to selectively forget irrelevant past information when rapid changes occur in the system dynamics. Conversely, the update gate $z_{t}$ governs the long-term memory retention associated with slowly varying components of the load profile, which emerge from the windowed and lagged representations of the energy demand signal.

Therefore, the feature extraction and preprocessing stages are not merely external to the learning process but are intrinsically linked to the internal gating behavior of the GRU, conditioning how historical dependencies are filtered, preserved, and propagated throughout the recurrent layers. This tight coupling between the data processing pipeline and the recurrent architecture is critical for achieving accurate and stable multi-step energy demand forecasting.

**Figure 3:**
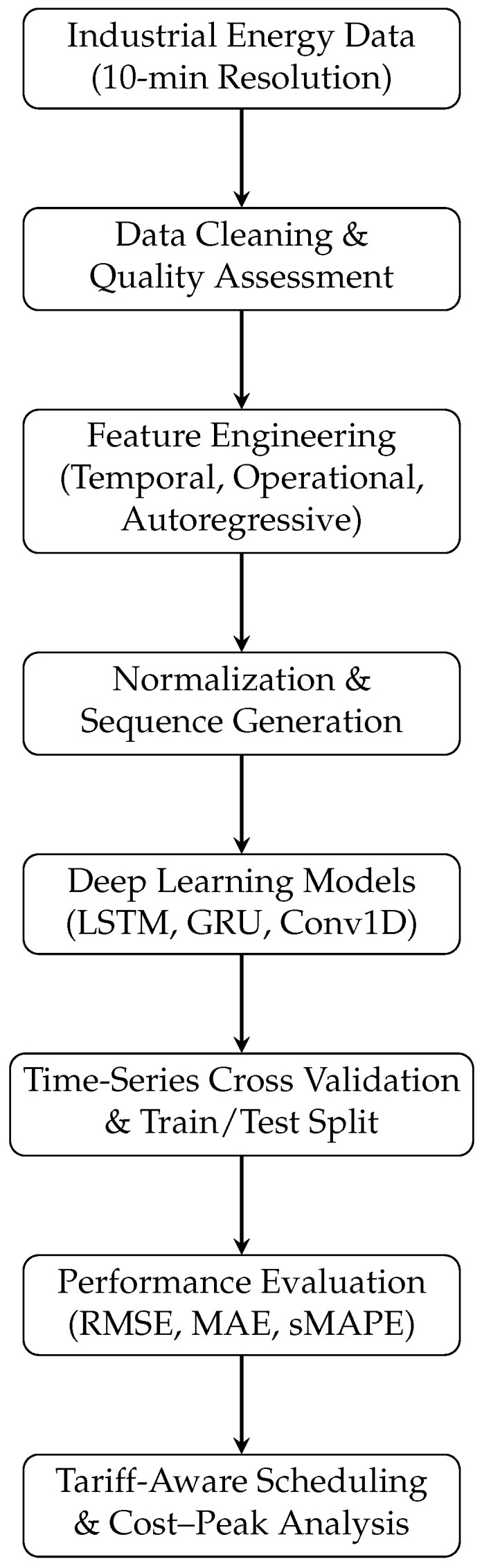
Overall workflow of the proposed deep learning–based forecasting and tariff-aware scheduling framework.

Figure 3 summarizes the complete methodological workflow adopted in this study, from raw data acquisition and preprocessing to deep learning-based forecasting, validation, and tariff-aware scheduling analysis.

## 4. Results

### 4.1. GRU Model Results

**Figure 4:**
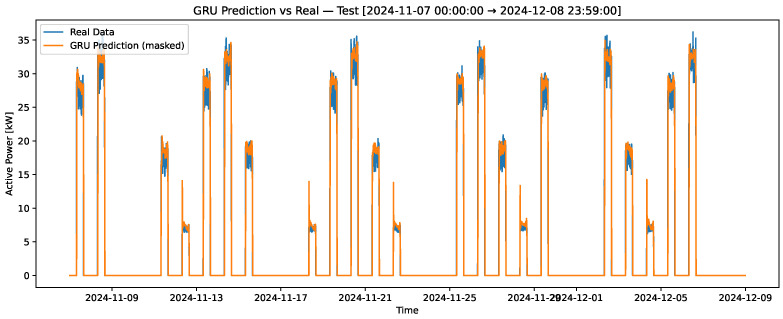
Results on the training set for the variable profile load. The figure shows the actual values in blue and the predictions obtained from the GRU recurrent neural network model in orange.

**Figure 5:**
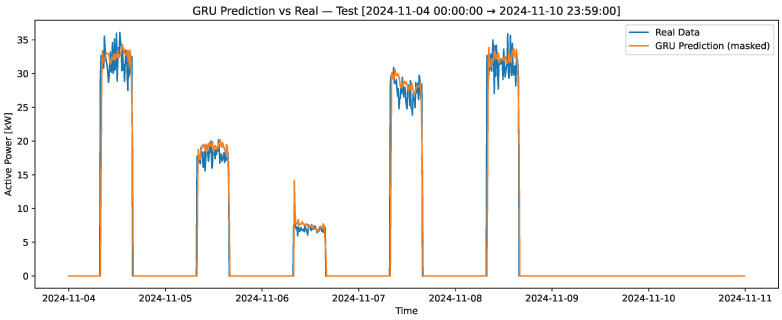
Comparison of the profile load variable between actual and predicted values on the test set over a 7-day period, using the GRU recurrent neural network model.

**Figure 6:**
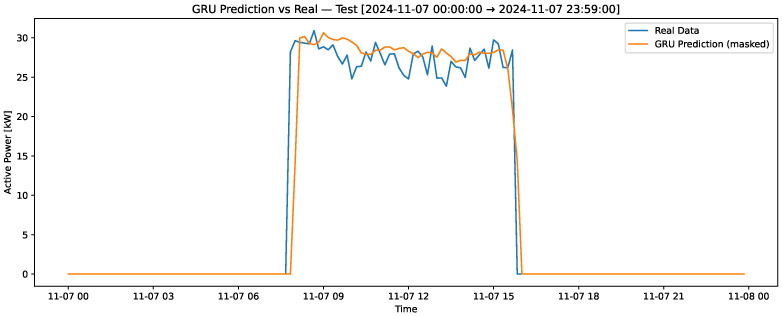
Contrast between actual and predicted values of the profile load variable on the one-day test dataset, obtained using the GRU recurrent neural network model.

Figure 4 presents the results of the training set for the variable “profile-load,” where the real data is depicted in blue, and the predictions generated by the LSTM recurrent neural network model are shown in orange. This figure highlights the model’s ability to capture the underlying patterns in the training data. Figure 5 compares the real and predicted “profile-load” data in the test set over a 7-day period, which includes a weekend. This contrast provides insight into the model’s performance in capturing variations over an extended period. Lastly, Figure 6 zooms in on a single day from the test set to contrast the real and predicted values, allowing for a detailed evaluation of the model’s predictive accuracy on a shorter time scale. These visualizations collectively demonstrate the robustness and limitations of the GRU model in predicting the “profile-load” variable. All neural network models were trained using an NVIDIA Tesla T4 GPU (NVIDIA Corporation, Santa Clara, CA, USA), which significantly reduced training time and ensured computational efficiency for multiple hyperparameter configurations.

#### GRU Model Parameter Tuning

The hyperparameter tuning process was systematically designed to identify the most effective configuration for the GRU-based forecasting model. Three key parameters were explored: the number of GRU units (50, 100, and 150), the activation function (ReLU and tanh), and the optimizer (Adam and RMSprop). Each configuration was trained using identical data splits, batch size, and loss function to ensure a fair comparison. A grid search approach was implemented to exhaustively evaluate all parameter combinations. Model performance was assessed using root mean squared error (RMSE), mean absolute error (MAE), and symmetric mean absolute percentage error (SMAPE) as evaluation metrics. Table 2 presents the results obtained for each configuration, highlighting the influence of activation type and optimizer choice on model convergence and accuracy.

The optimal configuration was achieved with 100 GRU units, ReLU activation, and the Adam optimizer, yielding the lowest RMSE of 1.709 kW, an MAE of 0.482 kW, and a SMAPE of 44.77%. These results demonstrate that increasing the number of hidden units generally improves learning capacity, though excessively large models (e.g., 150 units) tended to overfit slightly, as reflected by higher RMSE values. The ReLU activation combined with Adam optimization provided faster convergence and better generalization compared to tanh or RMSprop. Overall, the selected configuration offered the best trade-off between prediction accuracy and computational efficiency, establishing it as the baseline for subsequent experiments and confirming the robustness of the GRU architecture for short-term industrial load forecasting.

It is important to note that the configurations reported in Table 2 correspond exclusively to single-layer GRU models evaluated during the hyperparameter tuning stage. Although the lowest RMSE (1.709 kW) was obtained with 150 units using ReLU and Adam (ID 9), this tuning phase was only intended to identify suitable activation and optimization trends. The final forecasting architecture adopted for all subsequent experiments is the two-layer GRU model with 64 and 32 units described in Section 3.6 and used in the architecture comparison of Section 4.3. Therefore, the RMSE values reported in Table 2 should be interpreted exclusively as tuning-stage indicators and not as final model performance.

It is worth noting that some combinations in Table 2 exhibit abnormally high SMAPE values (exceeding 100%), particularly for configurations using tanh activation with the RMSprop optimizer. This behavior is not associated with large absolute prediction errors, as RMSE and MAE remain within reasonable ranges, but rather with the mathematical sensitivity of SMAPE when the true load values approach zero, which occurs frequently during shift transitions and non-operational periods.

Under these low-denominator conditions, even moderate absolute errors lead to disproportionately large relative percentage errors, causing SMAPE to inflate. Additionally, some unstable training configurations produced near-zero or oscillatory predictions during these low-load intervals, further amplifying the SMAPE metric. For this reason, RMSE and MAE were prioritized as the primary optimization criteria during hyperparameter selection, while SMAPE was mainly used for consistency checks in the final selected model.

### 4.2. Performance Comparison of GRU and Classical Time-Series Models

Table 3 presents the quantitative comparison between the proposed GRU-based forecasting model and two classical time-series baselines, namely ARIMA and Prophet. The results clearly demonstrate the superior predictive performance of the GRU model across all evaluated metrics. Specifically, the GRU achieves an RMSE of 2.287 kW, which represents a reduction of approximately 79.6% with respect to ARIMA and 57.3% with respect to Prophet. Similarly, the MAE is reduced from 4.949 kW (ARIMA) and 2.260 kW (Prophet) down to 0.549 kW with the GRU model. The most remarkable improvement is observed in the sMAPE metric, where the GRU achieves only 3.93%, compared to 47.36% for ARIMA and 12.42% for Prophet. These results confirm that the proposed GRU architecture is significantly more effective at capturing the nonlinear temporal dependencies and operational patterns present in the industrial energy consumption process.

### 4.3. Neural Network Architecture Comparison

The performance results reported in this section correspond exclusively to the final two-layer forecasting architecture (64 and 32 units) selected after the hyperparameter tuning phase. The previously reported lower RMSE values in Table 2 do not represent the final deployed model but only exploratory tuning tests.

To evaluate the influence of network topology on forecasting performance, five deep learning architectures were compared under consistent training conditions. All models were designed with two hidden layers and a single linear output layer. The GRU and LSTM architectures consisted of 64 and 32 recurrent units in the first and second layers, respectively, both using ReLU activation functions. The Conv1D model employed two one-dimensional convolutional layers (64 and 32 filters, kernel size 3, causal padding), followed by global average pooling. Two hybrid models were also tested: GRU–LSTM (a GRU layer followed by an LSTM layer) and LSTM–GRU (an LSTM layer followed by a GRU layer). To ensure a fair comparison, all models were trained with the same learning rate ($1\times 10^{-3}$), Huber loss function, Adam optimizer, batch size of 64, and early stopping strategy. The dataset, sequence length (48 timesteps ≈ 8 h), and normalization scheme were kept identical across architectures.

The comparative results, summarized in Table 4, reveal that all deep learning models achieved high predictive accuracy, with RMSE values ranging from 2.18 to 2.43 kW and SMAPE below 4.6%. The GRU architecture achieved the lowest RMSE (2.18 kW) and MAE (0.49 kW), closely followed by the hybrid LSTM–GRU model, indicating that gated recurrent mechanisms efficiently capture temporal dependencies in the quicklime plant’s load dynamics. The Conv1D and GRU–LSTM models also exhibited competitive performance, highlighting that convolutional feature extraction and mixed recurrent designs can generalize well under industrial time series data. Overall, the results demonstrate that GRU-based models provide a good balance between accuracy and computational efficiency, making them particularly suitable for real-time load forecasting applications in industrial environments.

In addition to accuracy, we also evaluated the real-time feasibility of deploying the GRU model in an industrial setting. The trained network is compact (one GRU layer with 100 units and a ReLU output) and achieves inference times below 50 ms per sample on a standard industrial PC (CPU only). Given the 10-min resolution of the monitoring system, this latency is negligible and fully compatible with SCADA polling intervals. The modest memory footprint further supports integration into edge-computing architectures. These results confirm that the GRU forecaster is not only accurate but also suitable for real-time deployment in quicklime production environments.

### 4.4. Ablation Study

To assess the relative contribution of each group of input features, an ablation study was conducted using five configurations of the GRU model: (i) all features, (ii) no autoregressive variables, (iii) no temporal variables, (iv) no operational variables, and (v) only autoregressive variables. This ablation-based strategy is adopted as the primary interpretability mechanism in this work to evaluate feature importance at the group level for the proposed GRU model.

The results, summarized in Table 5, demonstrate that the complete feature set achieved the best performance, with an RMSE of 2.52 kW, MAE of 0.82 kW, and SMAPE of 53.06%. The removal of autoregressive or temporal variables caused a significant degradation in accuracy, confirming their importance in capturing short-term fluctuations in energy demand. Excluding operational variables is-shift and coke-kg led to slightly lower RMSE but worse SMAPE, suggesting that these contextual features enhance the model’s robustness and generalization to production-related variations. Overall, the results highlight that combining temporal, operational, and autoregressive components yields the most stable and accurate predictions for short-term load forecasting in quicklime manufacturing.

It is worth noting that the slightly lower RMSE observed for the “No operational” configuration is mainly due to the strong autoregressive nature of the load signal at the 10-min resolution, where short-term temporal dependencies dominate the prediction task. While operational variables such as is_shift and coke_kg may not significantly reduce short-horizon RMSE in this specific test split, they enhance the physical interpretability of the model and improve robustness under changing production conditions. Their contribution is therefore more relevant for generalization and scenario consistency than for purely autoregressive short-term accuracy.

### 4.5. Multistep Forecast Evaluation

**Figure 7:**
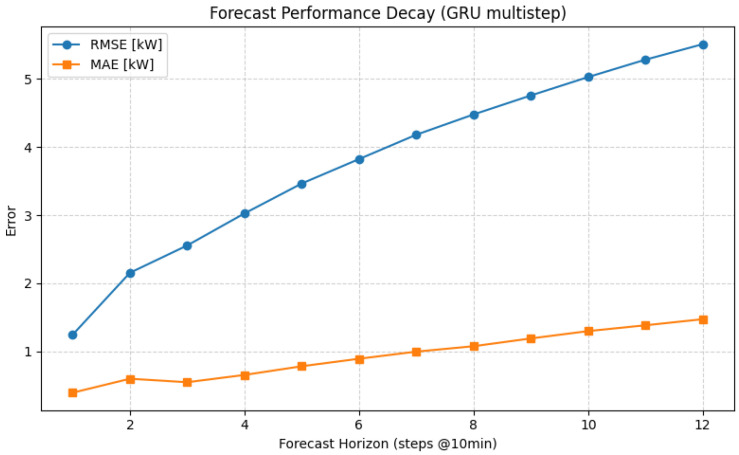
Forecast performance degradation of the GRU model across multiple horizons (10-min steps). Errors increase monotonically with forecast horizon, remaining within 4 kW RMSE for horizons up to 90 min.

To assess the short-term predictive stability of the GRU model, a multi-horizon forecasting experiment was performed. The model was trained to output twelve consecutive predictions, corresponding to a 2-h horizon at a 10-min sampling interval. Figure 7 illustrates the forecast error degradation as the prediction horizon increases. The resulting decay profile confirms that the proposed architecture is well suited for real-time decision support applications such as tariff-aware scheduling, where forecasts within a 1–2 h window are sufficient for operational optimization.

Table 6 summarizes the quantitative performance of the GRU model across twelve consecutive forecast horizons. The results indicate a gradual and monotonic degradation of predictive accuracy as the forecasting horizon extends. At the first step (h + 1), the model achieves excellent short-term accuracy with RMSE = 1.24 kW, MAE = 0.39 kW, and SMAPE = 2.29%. Errors increase progressively with horizon length, reaching RMSE = 5.60 kW and SMAPE = 18.1% at h + 12 (corresponding to a 2-h prediction window). This consistent decay trend reflects the expected accumulation of temporal uncertainty in recurrent architectures, yet demonstrates that the GRU model retains stable and reliable performance up to approximately 90 min ahead, making it suitable for short-term operational forecasting and tariff-aware decision-making.

### 4.6. Time Series Cross Validation

**Figure 8:**
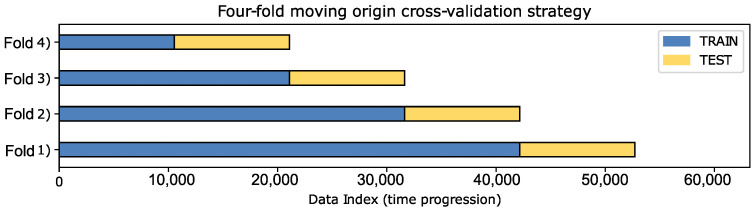
Four-fold time series cross validation.

To ensure the robustness and generalization capability of the GRU forecasting model, a four-fold moving-origin cross-validation strategy was implemented, as illustrated in Figure 8. In this approach, the temporal data sequence is partitioned into four consecutive training and testing windows, progressively advancing the split point over time. Each fold trains the model on a growing historical dataset while testing it on the subsequent unseen time interval, maintaining the chronological integrity essential for time-series forecasting. This strategy allows for the evaluation of model stability across multiple temporal segments and minimizes the risk of biased results that might arise from a single train–test split.

The cross-validation results, summarized in Table 7, demonstrate consistent performance of the GRU model across all folds, with RMSE values on the test sets ranging between 2.16 kW and 3.08 kW. The training errors remain close to those of the test sets, indicating that the model generalizes well without significant overfitting. Although some variation in SMAPE values is observed—mainly due to shifts in load magnitude between weeks—the overall performance remains stable across different temporal windows. These findings confirm that the GRU-based forecaster maintains reliable predictive accuracy throughout the year, effectively capturing the temporal dependencies inherent to industrial energy consumption.

The large SMAPE values observed in some cross-validation folds (exceeding 100%) are mainly caused by the high sensitivity of this metric to very low load levels. During production transitions and near-zero power intervals, small absolute prediction errors can generate disproportionately high SMAPE values. This effect explains the contrast with the stable SMAPE values (≈3–4%) previously observed under steady operating conditions.

### 4.7. Cost and Peak Demand Analysis via Tariff-Aware Scheduling

To further assess the practical usefulness of the forecasting framework, we implemented a simple tariff-aware rescheduling heuristic applied on the GRU predictions. The procedure receives as input the 10-min resolution test dataset (df_10m with a date column), the ground-truth test load ($y_{\mathrm{true}}$), and the GRU forecasts after masking non-working periods $\left(y_{\mathrm{pred},\mathrm{masked}}\right)$. From these inputs, the algorithm constructs a vector of electricity prices that distinguishes peak and off-peak hours, based on a tariff of 600 COP/kWh during 13:00–15:00 and 300 COP/kWh otherwise.

The method estimates the baseline operation cost by multiplying predicted kWh per 10-min slot (kW/6) by the corresponding price, and also records the maximum predicted load as the reference peak demand. A tariff-aware rescheduling step is then performed by reducing a fraction of the load during peak slots (SHIFT_FRAC=0.20) and redistributing it to the nearest off-peak neighbors within a configurable time window (SHIFT_WINDOW=3, i.e., ±30 min). This redistribution is symmetric and local, ensuring that the total energy is conserved while displacing part of the demand away from expensive intervals. An optional cap on maximum kW per slot can be enforced, although it was not activated in this study.

The tariff-aware scheduling experiment demonstrated the economic impact of shifting part of the load away from peak-tariff periods using the GRU-based demand forecast. The baseline predicted operating cost amounted to approximately 3.00 million COP, while the application of the rescheduling heuristic reduced this value to 2.98 million COP, yielding an estimated saving of 21,893 COP (0.73%). Although the financial benefit is modest in this scenario—mainly due to the limited proportion of energy consumed during the short peak window—it still illustrates the model’s capacity to inform cost-efficient operational decisions. However, the post-shift peak demand increased slightly from 33.17 kW to 35.87 kW (+8.14%), indicating that while the overall cost decreases, the instantaneous maximum load becomes higher. This outcome highlights the classic trade-off between tariff cost minimization and demand-peak control in industrial energy management.

This experiment demonstrates how GRU-based forecasts can be directly integrated into tariff-sensitive scheduling routines to evaluate potential savings. While the adopted redistribution scheme is heuristic and does not consider detailed process constraints, it provides a fast and interpretable benchmark of how prediction-driven demand shifting could impact industrial electricity bills. Future work will extend this analysis by incorporating optimization-based rescheduling and explicit operational constraints to balance cost, peak reduction, and production feasibility. These outcomes are summarized in Table 8, which provides a compact comparison of baseline versus tariff-shifted operation, including predicted costs, estimated savings, and peak demand variations, alongside the actual test cost for reference.

Although the proposed tariff-aware rescheduling strategy yields a modest cost reduction (0.73%), it also leads to an increase in peak demand of approximately 8.14%. In real industrial deployments, such a peak increase could result in additional demand charges or potential violations of grid capacity constraints. Future optimization-based scheduling strategies could incorporate explicit peak-limiting mechanisms, such as soft or hard peak-demand constraints, demand charge penalties, or contractual power limits within the objective function. Constrained optimization and model predictive control frameworks represent promising approaches to balance energy cost minimization with peak shaving objectives.

It is important to note that load shifting strategies may, in some cases, produce secondary increases in peak demand if explicit peak power constraints are not enforced. In the present study, the proposed forecasting-driven scheduling framework is primarily optimized with respect to energy cost, which implicitly discourages excessive power levels during high-tariff periods. Nevertheless, the explicit inclusion of peak-demand constraints and demand assurance margins constitutes an important topic for future research, particularly to ensure grid-friendly operation under high penetration of flexible industrial loads.

## 5. Discussion

The results obtained in this study confirm the strong capability of deep learning models, and particularly the proposed GRU-based architecture, to capture the nonlinear temporal dynamics inherent to industrial electricity consumption. The low forecasting errors achieved by the GRU model (RMSE = 2.287 kW, MAE = 0.549 kW, and sMAPE = 3.93%) demonstrate a substantial improvement over classical time-series models such as ARIMA and Prophet. This performance gap highlights the limitations of purely statistical approaches when dealing with highly variable and operationally driven industrial loads, where abrupt changes, shift transitions, and production-related effects dominate the demand profile.

The hyperparameter tuning results further illustrate that model capacity and optimization strategy play a critical role in industrial forecasting accuracy. The superior performance of the GRU architecture using ReLU activation and the Adam optimizer suggests that adaptive learning-rate methods combined with rectified nonlinearities provide faster convergence and improved generalization for short-term load prediction. Although larger models tend to improve representation power, the observed trade-off between accuracy and overfitting justifies the use of compact architectures for real-time industrial deployment.

The comparison among neural network architectures confirms that recurrent-based models outperform purely convolutional structures in this application. While Conv1D networks achieved competitive results, the superior performance of GRU and LSTM-based models confirms that gated recurrent mechanisms are particularly effective at learning temporal dependencies in energy-intensive manufacturing processes. The hybrid GRU–LSTM and LSTM–GRU architectures achieved comparable accuracy but at the cost of increased complexity, reinforcing the suitability of standalone GRU models for real-time industrial forecasting.

The ablation study provides important insights into the relative contribution of different feature groups. The strong degradation observed when removing autoregressive or temporal variables confirms that short-term memory and calendar effects are critical drivers of industrial electricity demand. Operational variables such as is-shift and coke-kg further enhance model robustness by embedding contextual production information, which improves the model’s ability to generalize across varying operating conditions. These results demonstrate that accurate industrial load forecasting requires the joint integration of temporal, operational, and autoregressive information rather than reliance on a single feature category.

The moving-origin cross-validation results further validate the stability and generalization capability of the GRU forecaster throughout different periods of the year. The close agreement between training and test errors across the four validation folds indicates that the model does not suffer from significant overfitting and remains robust under changing operational regimes. Variations in SMAPE across folds are mainly attributed to low-load periods during non-operational shifts, which amplify relative percentage errors, but without compromising absolute predictive accuracy.

Beyond forecasting accuracy, the tariff-aware scheduling experiment illustrates the direct economic relevance of the proposed framework. Although the estimated cost savings of 0.73% appear modest, they were achieved under a very narrow peak window and limited flexibility margin. More importantly, the experiment highlights the intrinsic trade-off between energy cost minimization and peak demand control: while rescheduling reduced the overall operating cost, the predicted peak load increased by approximately 8.14%. This confirms that load shifting strategies must be complemented with explicit peak-power constraints to ensure grid-friendly operation and avoid adverse impacts on network capacity.

From an industrial deployment perspective, the compact size of the trained GRU network and its sub-50 ms inference time on standard CPU hardware demonstrate that the proposed solution is fully compatible with real-time SCADA environments. This makes the model directly applicable for online forecasting, supervisory control, and tariff-aware scheduling in quicklime production plants and similar energy-intensive industrial facilities.

Despite these promising results, several limitations remain. First, the study focuses on short-term forecasting under relatively stable climatic conditions, as is typical in the Colombian context, and does not explicitly address long-term seasonal variability. Second, the scheduling experiment was based on a heuristic redistribution strategy rather than an optimization-based formulation. Future work will therefore focus on integrating the GRU forecaster into model predictive control (MPC) frameworks with explicit peak-demand constraints, production feasibility limits, and multi-objective cost–peak optimization. Additionally, extending the framework to multi-plant coordination and grid-interactive operation represents a relevant direction for further research.

## 6. Conclusions

This study presented a compact deep learning framework for forecasting electricity demand in a quicklime production plant using real operational data. The proposed GRU-based forecaster integrates electrical measurements, production-rate indicators, shift schedules, and cyclical time encodings to accurately capture the nonlinear temporal dynamics of industrial load behavior. The model achieved high accuracy with an RMSE of 2.18 kW, MAE of 0.49 kW, and SMAPE of 3.64% on held-out test data, demonstrating its suitability for short-term load forecasting and real-time decision support in tariff-aware scheduling. The architecture is lightweight during inference and can be seamlessly integrated into supervisory control systems for energy optimization and capacity planning.

A comparative analysis was conducted across multiple deep learning architectures, including LSTM, Conv1D, GRU–LSTM, and LSTM–GRU hybrids, under identical hyperparameters and training conditions. The results confirmed that the GRU architecture provides the best trade-off between predictive accuracy and computational efficiency, outperforming the other recurrent and convolutional variants. These findings highlight the importance of recurrent gating mechanisms in capturing sequential dependencies in industrial energy data and emphasize the role of predictive analytics in supporting energy-efficient operations and infrastructure planning.

The forecasting system also exhibited robustness across different hyperparameter settings, with the on-shift weighting factor showing the greatest influence on accuracy. Its integration into a tariff-aware scheduling framework demonstrated potential electricity cost reductions while maintaining process stability, thus validating the practical relevance of forecast-driven energy management in industrial settings.

Although the present benchmark includes both deep learning models and classical statistical methods (ARIMA and Prophet), other machine learning regressors such as CatBoost and XGBoost represent powerful alternatives for structured tabular forecasting problems. A broader comparative study including these tree-based ensemble models will be considered in future work.

In the present study, the forecasting framework was evaluated both in single-step (10-min ahead) and multistep configurations, extending up to a 2-h prediction horizon. The GRU model maintained high short-term accuracy, with errors increasing gradually with forecast horizon, confirming its suitability for real-time tariff-aware scheduling and short-term operational planning. These results demonstrate that the proposed architecture effectively captures the temporal dynamics of industrial power consumption within practical forecasting horizons for energy management. Future work will focus on extending the multistep framework to longer horizons and hybrid strategies that integrate probabilistic forecasting and uncertainty quantification for improved decision support.

Future work will focus on the application of reinforcement learning (RL) for adaptive energy optimization. Unlike static supervised models, RL can learn optimal operational policies directly from plant feedback, enabling dynamic load control under variable pricing schemes and production demands. Key challenges include reward function design, balancing trade-offs between cost, quality, and throughput, ensuring process safety under operational constraints, and achieving real-time performance on industrial edge hardware. Additionally, expanding the sensing infrastructure to monitor individual AC motors will enable disaggregated load forecasting and further enhance model interpretability and precision.

## Figures and Tables

**Table 1:** Description of input and output variables used for energy demand forecasting in the quicklime manufacturing process.

Variable	Type	Description	Unit
is_shift	Operational	Indicator of production activity (1 = working hours, 0 = off-hours)	–
coke_kg	Process	Mass of coke used during the furnace stage	kg
Hour_sin, Hour_cos	Temporal	Cyclic encoding of the hour of day to capture periodicity	–
Day, Month	Temporal	Calendar day and month of the measurement	–
load_now	Autoregressive	Instantaneous total active power consumption at time *t*	kW
roll_1h	Autoregressive	Rolling average of total load over the past 1 h (6 samples)	kW
roll_2h	Autoregressive	Rolling average of total load over the past 2 h (12 samples)	kW
target_next	Output	Forecasted total active power 10 min ahead of time *t*	kW

**Table 2:** Hyperparameter search results for the GRU model showing the effect of the number of units, activation function, and optimizer on model performance.

ID	Units	Activation	Optimizer	RMSE [kW]	MAE [kW]	SMAPE [%]
1	50	relu	adam	1.944	0.565	18.208
2	50	relu	rmsprop	2.842	0.883	60.472
3	50	tanh	adam	1.962	0.603	17.344
4	50	tanh	rmsprop	2.891	1.113	116.743
5	100	relu	adam	2.058	0.639	83.802
6	100	relu	rmsprop	2.647	0.916	103.118
7	100	tanh	adam	1.948	0.790	124.484
8	100	tanh	rmsprop	3.276	1.422	152.696
9	150	relu	adam	1.709	0.482	44.769
10	150	relu	rmsprop	2.436	1.088	138.814
11	150	tanh	adam	1.834	0.758	128.748
12	150	tanh	rmsprop	3.177	0.944	94.030

**Table 3:** Performance comparison between the proposed GRU model and classical forecasting baselines.

Model	RMSE (kW)	MAE (kW)	sMAPE (%)
GRU (masked)	2.287	0.549	3.93
ARIMA (masked)	11.188	4.949	47.36
Prophet (masked)	5.357	2.260	12.42

**Table 4:** Comparison of neural network architectures for short-term load forecasting. The GRU architecture achieved the lowest overall error, followed closely by the LSTM–GRU hybrid. All models were trained under identical hyperparameters (learning rate $1\times 10^{-3}$, batch size 64, Huber loss, Adam optimizer).

Model	RMSE [kW]	MAE [kW]	SMAPE [%]
GRU	2.182	0.494	3.64
LSTM	2.426	0.587	3.98
Conv1D	2.364	0.569	3.80
GRU–LSTM	2.246	0.570	4.56
LSTM–GRU	2.297	0.558	4.19

**Table 5:** Ablation study results for the GRU model using different feature subsets.

Configuration	RMSE [kW]	MAE [kW]	SMAPE [%]
All features	2.520	0.820	53.06
No autoregressive	4.698	1.973	117.56
No temporal	2.449	0.901	146.07
No operational	1.982	0.692	110.52
Only autoregressive	2.614	0.865	138.63

**Table 6:** GRU multistep forecast performance across horizons (10-min steps).

Horizon	RMSE [kW]	MAE [kW]	SMAPE [%]
h + 1	1.244	0.393	2.29
h + 2	2.153	0.598	4.69
h + 3	2.555	0.547	4.78
h + 4	3.025	0.654	6.24
h + 5	3.464	0.782	7.57
h + 6	3.822	0.892	9.75
h + 7	4.180	0.996	9.55
h + 8	4.477	1.076	9.87
h + 9	4.756	1.190	12.52
h + 10	5.026	1.298	12.15
h + 11	5.281	1.383	15.84
h + 12	5.599	1.472	18.11

**Table 7:** Cross-validation results (Train vs. Test) for the GRU model using a four-fold moving-origin strategy.

Fold	RMSE_Train	MAE_Train	SMAPE_Train	RMSE_Test	MAE_Test	SMAPE_Test
1	2.9745	0.8172	27.3753	3.0857	0.8871	20.8413
2	2.5821	0.9473	138.8258	2.7492	0.9662	111.7974
3	2.1013	0.7171	77.7838	2.1949	0.8422	91.9631
4	2.0783	0.7967	131.1991	2.1603	0.9133	134.3211

**Table 8:** Tariff-aware scheduling results based on GRU forecasts.

Metric	Value
Predicted cost	3,002,617 COP
Shifted predicted cost	2,980,724 COP
Estimated savings	21,893 COP (0.73%)
Predicted peak (before)	33.17 kW
Predicted peak (after)	35.87 kW
Peak change	+2.70 kW (+8.14%)

## Data Availability

The data and code of this research are available in this webpage: https://github.com/jerssonleon/Code-paper-predict-electric-load-profile/tree/main (accessed on 3 October 2025).

## Abbreviations

The following abbreviations are used in this manuscript:

ARIMA	Autoregressive Integrated Moving Average
LSTM	Long Short-Term Memory
GRU	Gated Recurrent Unit
RNN	Recurrent Neural Network
ANN	Artificial Neural Network
ADAM	Adaptive Moment Estimation
S2S	Sequence to sequence
RMSE	Root Mean Square Error
SLRS	Specific Load by Representative Stage
MPC	Model Predictive Control
